# Agronomic Performance and Nitrogen Fixation of Heirloom and Conventional Dry Bean Varieties Under Low-Nitrogen Field Conditions

**DOI:** 10.3389/fpls.2019.00952

**Published:** 2019-07-26

**Authors:** Jennifer Wilker, Alireza Navabi, Istvan Rajcan, Frédéric Marsolais, Brett Hill, Davoud Torkamaneh, K. Peter Pauls

**Affiliations:** ^1^Department of Plant Agriculture, University of Guelph, Guelph, ON, Canada; ^2^Agriculture and Agri-Food Canada, London Research and Development Centre, London, ON, Canada; ^3^Agriculture and Agri-Food Canada, Lethbridge Research and Development Centre, Lethbridge, AB, Canada

**Keywords:** nitrogen fixation, symbiosis, heirloom, bean, breeding

## Abstract

Common beans (*Phaseolus vulgaris*) form a relationship with nitrogen-fixing rhizobia and through a process termed symbiotic nitrogen fixation (SNF) which provides them with a source of nitrogen. However, beans are considered poor nitrogen fixers, and modern production practices involve routine use of N fertilizer, which leads to the down-regulation of SNF. High-yielding, conventionally bred bean varieties are developed using conventional production practices and selection criteria, typically not including SNF efficiency, and may have lost this trait over decades of modern breeding. In contrast, heirloom bean genotypes were developed before the advent of modern production practices and may represent an underutilized pool of genetics which could be used to improve SNF. This study compared the SNF capacity under low-N field conditions, of collections of heirloom varieties with and conventionally bred dry bean varieties. The heirloom-conventional panel (HCP) consisted of 42 genotypes from various online seed retailers or from the University of Guelph Bean Breeding program seedbank. The HCP was genotyped using a single nucleotide polymorphism (SNP) array to investigate genetic relatedness within the panel. Field trials were conducted at three locations in ON, Canada from 2014 to 2015 and various agronomic and seed composition traits were measured, including capacity for nitrogen fixation (using the natural abundance method to measure seed N isotope ratios). Significant variation for SNF was found in the panel. However, on average, heirloom genotypes did not fix significantly more nitrogen than conventionally bred varieties. However, five heirloom genotypes fixed >60% of their nitrogen from the atmosphere. Yield (kg ha^-1^) was not significantly different between heirloom and conventional genotypes, suggesting that incorporating heirloom genotypes into a modern breeding program would not negatively impact yield. Nitrogen fixation was significantly higher among Middle American genotypes than among Andean genotypes, confirming previous findings. The best nitrogen fixing line was Coco Sophie, a European heirloom white bean whose genetic makeup is admixed between the Andean and Middle American genepools. Heirloom genotypes represent a useful source of genetics to improve SNF in modern bean breeding.

## Introduction

Since its origin in central Mexico some 2 My ago, common bean (*Phaseolus vulgaris* L.) has diverged into two genepools in Central America and South America, been domesticated and spread throughout the world (Kaplan and Lynch, 1999; Gepts et al., 2000; Bitocchi et al., 2017). First Nations’ ancestral groups gathered wild beans and cultivated them with other crops, including maize (*Zea* spp.) and squash (*Cucurbita* spp.). Beans were among the crops which explorers brought back to Europe after they visited the Americas. Centuries of cultivation and movement of seed through human migration and trade led to beans becoming staples in diets around the world, and inseparable parts of numerous cultural heritages. Recent years have seen increases in heirloom bean popularity, stretching beyond farmers’ markets and seed exchanges to specialty grocers, culinary circles, and mainstream culture.

Before the establishment of formal bean breeding programs, landraces maintained by First Nations groups and European settlers were grown throughout North America (Kelly, 2010). Aside from their historical origin and association with early farming systems, bean landraces are characterized by having local genetic adaptation, high genetic diversity and a lack of formal genetic improvement (Villa et al., 2005). In many instances, heirloom beans have distinctive characteristics such as unique seed coat colors/patterns, and desirable flavors or cooking traits. However, yield, disease resistance, and growth habit may be poor compared to conventionally bred, relatively modern, bean cultivars. In contrast, modern bean cultivars conform to standard requirements for size and color particular to a few market classes, and are bred to produce high yields under conventional production practices (Kelly, 2010). Market demands and producer requirements are believed to have led to narrow breeding objectives and reduced genetic diversity in modern bean cultivars (Singh, 1988). This reduction in genetic diversity may have also led to a reduction in diversity and capacity for nitrogen fixation in modern bean genotypes.

Between the two genepools of common bean, the Andean genepool is much less diverse than the Middle American genepool. This reduced diversity is a result of a bottleneck created when founder populations established the Andean genepool at a distance from the center of origin of bean, in present-day central Mexico (Bitocchi et al., 2012). The independent and parallel domestication of beans beginning some 8000 years ago in the Andean and Middle American regions resulted in separate genepools of domesticated bean (Papa and Gepts, 2003; Chacón et al., 2005; Kwak and Gepts, 2009; Rossi et al., 2009; Mamidi et al., 2011; Nanni et al., 2011; Bitocchi et al., 2013, 2017; Schmutz et al., 2014; Rendón-Anaya et al., 2017). The divergence has led to some difficulties in hybridization between Andean and Middle American genotypes (Johnson and Gepts, 1999). Nevertheless, introgression between genepools has been found in bean collections throughout the world (Gioia et al., 2013). In particular, introgression has influenced the diversity of the bean germplasm grown across Europe, where 40.2% of accessions show introgression compared to the much lower level of introgression in North American genotypes, which is 12.3% (Gioia et al., 2013).

Symbiotic nitrogen fixation (SNF) is an ancient trait, characteristic of the Fabaceae family. In bean, *Rhizobium leguminosarum* bv *phasioli* bacteria inhabit root nodules and fix atmospheric nitrogen, which is utilized by the plant in exchange for carbohydrates. However, among modern leguminous crops, beans are considered to be poor nitrogen fixers (Hardarson et al., 1993). In the latter half of the 20th century, research largely concluded that rates of nitrogen fixation in bean were low, at 25 to 71 kg N_2_ fixed ha^-1^ for mid- to long-season cultivars (Graham, 1981). These values are considerably lower than rates for soybean at the time, which ranged from 78 to 161 fixed ha^-1^ in one study (Muldoon et al., 1980). LaRue and Patterson (1981) reviewed multiple studies of nitrogen fixation in legume species and calculated that soybean fixed 75 kg N_2_ ha^-1^ on average while dry beans fixed just 10 kg N_2_ ha^-1^. However, recent studies have examined hundreds of bean genotypes for traits related to nitrogen fixation and reported wide-ranging capacity for these traits (Ramaekers et al., 2013; Kamfwa et al., 2015; Diaz et al., 2017; Farid et al., 2017; Heilig et al., 2017; Wilker et al., unpublished), indicating genotypic and genetic diversity which could be exploited to enhance this trait through breeding. For example, Farid (2015) tested twelve modern genotypes and found nitrogen fixing capacity ranged from 2.7 to 69.7 kg N_2_ fixed ha^-1^, which represents a range of 5.2 to 78.5% nitrogen derived from the atmosphere (%Ndfa). Heilig (2015) examined 79 navy and black commercial cultivars and advanced breeding lines under organic production and found a similar range for nitrogen fixing capacity (16 to 94 kg N_2_ ha^-1^) and for %Ndfa (9.8 to 71.7%).

Nitrogen fixation and root nodule traits are controlled by multiple genes. They are affected by environmental conditions, and are difficult to measure. As a result, modern bean breeding programs do not focus on breeding genotypes efficient at nitrogen fixation but rather release high-yielding genotypes which perform consistently under conventional production practices, which include the application of 33–67 kg ha^-1^ of nitrogen fertilizer (OMAFRA, 2009) and crop protection chemicals. In contrast, many heirloom varieties were developed and are maintained under natural growing conditions where fertility is managed using crop rotation and organic fertilizer and symbiosis with appropriate *Rhizobia* species occurs naturally or is enhanced by the use of inoculants. Therefore, heirloom genotypes may be a genetic resource for modern breeding programs that contain genetic diversity for nitrogen fixation and other traits that have not been eroded by modern breeding practices.

Nitrogen fixation capacity among modern dry bean varieties needs to be improved and discovery of diversity for the trait will provide genetic resources for breeding programs. The current study tests the hypothesis that heirloom beans have a greater capacity for nitrogen fixation than conventionally bred bean varieties and examines whether they could be useful germplasm sources to improve this trait. The objectives of this study were to compare heirloom and conventionally bred bean genotypes from both the Andean and Middle American genepools for their capacity for SNF, to assess whether genetic diversity has been lost over years of modern breeding, and to assess agronomic characteristics to determine the suitability of using heirloom varieties in modern breeding programs.

## Materials and Methods

### Plant Material

**FIGURE 1:**
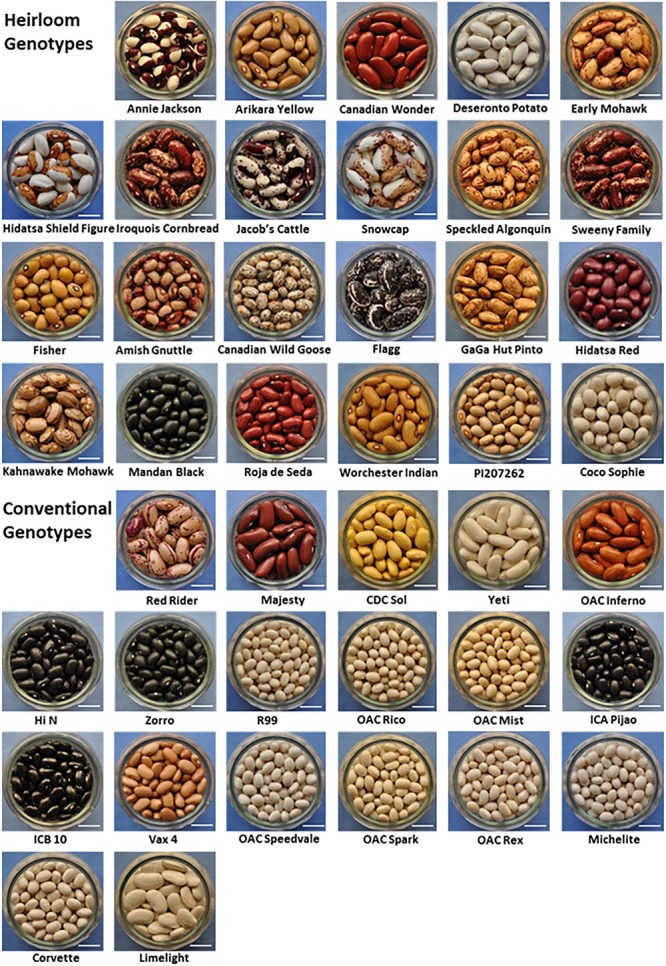
Images of *Phaseolus vulgaris* genotypes included in the heirloom-conventional panel. Twenty-three heirloom bean genotypes and nineteen conventionally bred bean genotypes grown at Elora and Belwood, Ontario, 2015 are shown. White bar = 1cm.

The heirloom-conventional panel (HCP) was assembled in 2014 and contained 25 heirloom and 17 conventionally bred dry bean genotypes. In the first growing season, six genotypes failed to reach physiological maturity and were removed from the panel. For the second growing season, six new genotypes were added, and the HCP consisted of 23 heirloom and 19 conventional genotypes. Only genotypes for which two or three location years of data was collected are included in the analyses in this report. Seed images of the genotypes in the HCP are displayed in Figure 1.

Heirloom seeds were purchased as pure line varieties from Canadian on-line seed retailers (Heritage Harvest Seed^1^, Assiniboine Tipis^2^, and Annapolis Seeds^3^) with the intent of including a wide representation of seed coat patterns, seed sizes and plant growth habits. Heirloom seed coat patterns ranged from uniform, to bi-color spotted/speckled/striped, or tri-color; often very different in appearance compared to conventional market classes. In this study, the term “heirloom” refers to genotypes of the HCP that were not derived from a conventional bean breeding program. Given the limited information available for each heirloom genotype in this panel (see compiled variety descriptions^4^), it was impossible to further categorize these genotypes into groupings such as “improved landrace” or “vintage cultivar.”

Seed of conventional bean genotypes was sourced from the University of Guelph Bean Breeding program’s seed stores. Germplasm was chosen to represent a range of market classes, seed sizes and growth habits, mirroring the diversity found among the heirloom genotypes, where possible. Conventional genotypes were registered with the Canadian Food Inspection Agency between 1938 and 2016 and were developed by modern breeding programs and institutions [including: University of Guelph (UG), Michigan State University (MSU), United States Department of Agriculture-Agriculture Research Station (USDA-ARS), Crop Development Centre (CDC) in Saskatchewan, International Center for Tropical Agriculture (CIAT), Instituto Colombiano Agropecuario (ICA), and Agriculture Agrifood Canada (AAFC) in Ontario and Alberta]. Descriptions of the genotypes, including market class, origin, seed size, plant growth habit, and genepool membership are presented for the HCP in Table 1.

**Table 1:** Market class, seed size, growth habit, genepool and race for 42 dry bean (*Phaseolus vulgaris* L.) genotypes of the heirloom-conventional panel.

Cultivar code	Cultivar name	Market class	Origin, year of CFIA registration	Seed size^†^	Growth habit¶	Gene pool^‡^	References
	**Heirloom**						
2	Annie Jackson	Red calypso	Russian heirloom, na	Medium	III	Andean	Heritage Harvest Seed
3	Arikara Yellow	Canario mexicano	Arikara FN, 2002	Medium	I	Andean	Mündel et al., 2004
5	Canadian Wonder	Red kidney	unknown, na	Large	I	Andean	Heritage Harvest Seed
7	Deseronto Potato	White kidney	Mohawk FN, na	Large	II	Andean	Heritage Harvest Seed
8	Early Mohawk	Cranberry	Iroquois FN, na	Large	I	Andean	Assiniboine Tipis
13	Hidatsa Shield Figure	Unknown	Hidatsa FN, na	Large	II	Andean	Heritage Harvest Seed
15	Iroquois Cornbread	Speckled red kidney	Iroquois FN, na	Large	I	Andean	Heritage Harvest Seed
17	Jacob’s Cattle	Unknown	Unknown, na	Large	I	Andean	Heritage Harvest Seed
21	Snowcap	Unknown	Unknown, na	Large	II	Andean	Heritage Harvest Seed
22	Speckled Algonquin	Cranberry	Algonquin FN, na	Medium	I	Andean	Heritage Harvest Seed
23	Sweeney Family	Speckled red kidney	Canadian heirloom, na	Large	I	Andean	Heritage Harvest Seed
25	Worchester Indian	Tan	Unknown, na	Medium	I	Andean	Heritage Harvest Seed
46	Coco Sophie	Navy	French heirloom, na	Medium	III	Andean	Heritage Harvest Seed
47	Fisher	Tan	Algonquin FN, na	Medium	I	Andean	Assiniboine Tipis
1	Amish Gnuttle	Unknown	Seneca FN, na	Small	III	MA	Annapolis Seeds
4	Canadian Wild Goose	Gray speckle	unknown, na	Small	II	MA^∗^	Heritage Harvest Seed
10	Flagg	Speckled black kidney	Iroquois FN, na	Large	III	MA	Assiniboine Tipis
11	Ga Ga Hut Pinto	Pinto	Seneca FN, na	Medium	II	MA	Heritage Harvest Seed
12	Hidatsa Red	Small red	Hidatsa FN, na	Medium	II	MA	Heritage Harvest Seed
16	Kahnawake Mohawk	Pinto	Mohawk FN, na	Large	III	MA^∗^	Annapolis Seeds
18	Mandan Black	Black	Mandan FN, na	Small	II	MA	Heritage Harvest Seed
20	Roja de Seda	Small red	Central American heirloom, na	Small	III	MA	Heritage Harvest Seed
36	PI207262	Tan	Gene bank plant introduction, na	Small	II	MA	Coyne and Schuster, 1974
	**Conventional**						
26	Red Rider	Cranberry	AAFC, 2008	Large	I	Andean	Park et al., 2009
27	Majesty	Red kidney	AAFC, 2005	Large	II	Andean	CFIA1, 2006; Park, 2006
28	CDC Sol	Yellow	CDC, 2010	Medium	I	Andean	CFIA2, 2013; Vandenberg and Bett, 2013
29	Yeti	White kidney	UG, 2013	Large	I	Andean	Khanal et al., 2016
43	OAC Inferno	Light red kidney	UG, 2011	Large	I	Andean	Smith et al., 2012
9	Hi N line	Black	UG, na	Small	II	MA	Breeding line
30	Zorro	Black	MSU, 2012	Small	II	MA	Kelly et al., 2009
32	R99	Navy	AAFC, na	Small	II	MA^∗^	Park and Buttery, 2006
33	OAC Rico	Navy	UG, 1983	Small	II	MA	Beversdorf, 1984
34	Mist	Navy	UG, 2013	Small	II	MA	Khanal et al., 2017a
35	ICA Pijao	Black	ICA, na	Small	II	MA	Voysest, 2000
37	ICB-10	Black	USDA-ARS, na	Small	II	MA	Miklas et al., 1999
38	VAX 4	Tan	CIAT, na	Small	II	MA	Singh et al., 2001
39	OAC Speedvale	Navy	UG, 1991	Small	II	MA	CFIA3, 1991
41	OAC Spark	Navy	UG, 2012	Small	I	MA	Khanal et al., 2017b
42	OAC Rex	Navy	UG, 2002	Small	II	MA	Michaels et al., 2006
44	Michelite	Navy	MSU, 1940	Small	II	MA	Kelly, 2010
45	Corvette	Navy	AAFC, 1943	Small	II	MA	McGregor, 1956
48	Limelight	Navy/wt kidney	AAFC, 1972	Medium	I	MA	Sears, 1986

*^†^Small, 13 to 29 g per 100 seeds; medium, 30 to 45 g per 100 seeds; large, 46 to 63 g per 100 seeds. ^¶^ Growth habit according to Singh (1982). ^‡^Genepool assigned according to STRUCTURE analysis. Threshold genetic contribution from assigned genepool was >50%. Genotypes marked with (^∗^) were assigned to genepool according to market class appearance – these genotypes were not SNP genotyped MA, Middle American.*

### Field Experimental Design and Maintenance

Field trial locations were selected based on low soil nitrogen levels as measured by pre-planting soil tests which showed that rate levels of NO_3-_ were under 5 ppm (“very low”) or 5–10 ppm (“low”) and by site crop rotation histories that indicated that no dry bean crops had been produced at the sites for the previous decade, at a minimum. Soil nitrogen and growing season details can be found in Supplementary Table 1.

Clean seed of each genotype was coated with commercially available Nodulator (Becker-Underwood) *Rhizobium legumin osarum* bv *phaseoli* inoculant prior to planting. The day before planting, 1/8 teaspoon (approximately 0.2 g) of inoculant powder was added to each seed envelope and the contents were shaken to coat the seeds. Inoculated seed was stored at the Elora Research Station (ERS) at 4°C until planting to maintain inoculant viability. The entire contents of each envelope (coated seed + loose inoculant powder) was planted.

The HCP was grown in three low-nitrogen field location-years using a rectangular lattice design (6 × 7) with two replications. At the ERS in 2014, 100 seeds of each genotype were grown in single-row plots 6 m in length with approximately 6 cm between plants and 60 cm spacing between entry rows. In 2015, the HCP was grown in another field at the ERS and at an offsite location near Belwood, Ontario. Increased seed availability enabled planting of 135 seeds in 4-row plots (150 cm × 90 cm, 37.5 cm between rows) with approximately 5 cm between plants within rows.

Throughout the growing season, plots were maintained with standard practices, except no-nitrogen fertilizer was used. Pre-plant fertilizer (0–20–20) at a rate of 200 kg ha^-1^ was applied approximately 1 week prior to planting. Pre-plant herbicides [200 ml ha^-1^ Pursuit (BASF) and 1.5 L ha^-1^ Frontier (BASF)] were applied to control broadleaf and grass weeds. At Elora 2014, insecticides against leaf hoppers were applied July 11 [1.0 L ha^-1^ Lagon (Loveland products) and 40 ml ha^-1^ Matador (Syngenta)], fungicides against Anthracnose and root rot were applied July 11 [0.5 L ha^-1^ Quadris (Syngenta) and 1.0 L ha^-1^ Allegro (Syngenta)], and again against Anthracnose on August 7 [400 ml ha^-1^ Headline (BASF) and 1 L ha^-1^ Allegro]. At Elora 2015, herbicides were applied July 15 [2.25 L ha^-1^ Basagran (BASF) and 0.67 L ha^-1^ Excel Super (Excel Crop Care)], [1 L ha^-1^ Assist (BASF)] followed by insecticides against leaf hoppers [1.0 L ha^-1^ Cygon (FMC Corporation) and 40 ml ha^-1^ Matador] and fungicides against Anthracnose [400 ml ha^-1^ Headline (BASF) and 1 L ha^-1^ Allegro] on July 16. Fungicide against Anthracnose (0.5 L ha^-1^ Quadris) and insecticide against leaf hoppers [200 ml ha^-1^ Admire (Bayer)] were again applied August 6. At Belwood 2015, insecticides (1.0 L ha^-1^ Cygon and 40 ml ha^-1^ Matador) and fungicides (400 ml ha^-1^ Headline and 1 L ha^-1^ Allegro) were applied on July 16. The Belwood plots were treated against Anthracnose (0.5 L ha^-1^ Quadris) and leaf hoppers (200 ml ha^-1^ Admire) again on August 6. Plots at all locations were manually weeded once before canopy closure each year.

### Phenotyping

Days to flowering was observed throughout July and August and was recorded as the date when 50% of the plants in a plot had one flower open. The days to flowering measurements were converted into growing degree days to flowering (GDDf) by summing the calculated GDD temperature from daily max and min temperatures. Hourly temperatures were recorded at the ERS by the University of Guelph School of Environmental Sciences Agrometeorology group^5^. For the Belwood site, temperature data from the nearest Government of Canada weather station data was used (Fergus Shand Dam^6^).

Relative leaf chlorophyll content was measured twice during the growing season [when the mean number of plots had reached (1) the second trifoliate stage, and (2) at 100% flowering] using a SPAD 502 Plus Chlorophyll Meter (Konica Minolta). The meter was calibrated according to manufacturers’ instructions each time the unit was powered-on^7^. The middle leaflet in the top-most, fully expanded trifoliate leaf was used for the measurements and three plants were sampled per plot.

Plots were rated for days to maturity throughout September and early October. Plots were considered to have reached maturity when they were ready for harvest. Days to maturity measurements were converted into growing degree days to maturity (GDDm) in the same way as for GDDf (see above).

Three plants were randomly sampled from mature plots, placed in large paper bags, and dried in a re-purposed tobacco kiln (De Cloet Bulk Curing Systems, model TPG-360, Tillsonburg, ON, Canada) at 33°C at the ERS for 24–48 h. Prior to weighing, roots were cut from each plant and above-ground biomass was measured. Plants were then threshed using an indoor belt thresher (Agriculex SPT-1A, Guelph, ON, Canada), their seed collected, weighed and counted. Harvest index (biomass/seed weight) as well as 100 seed weight (HSW) were calculated.

At Elora 2014, the harvest was staggered according to maturity. The plots were pulled by hand at maturity and threshed at the side of the field using a Wintersteiger plot combine (Wintersteiger AG, Upper Austria, Austria) with a Classic Seed-Gauge weighing system by Harvest-Master (Juniper Systems Inc., UT, United States) and plot seed weight and moisture content were recorded. In 2015, plot harvest took place after all plots reached maturity with the same Wintersteiger combine.

### Seed Isotope Analysis

The natural abundance method (Shearer and Kohl, 1986) was used to calculate percent nitrogen derived from the atmosphere (%Ndfa) for each genotype. Seed was used for this assessment because seed N at maturity represents the total N accumulated over the growing season, whereas shoot N is transitory and fluctuates over the plant life cycle (Masclaux-Daubresse et al., 2010) making coordination of sampling times challenging in studies with multiple genotypes. Additionally, %Ndfa levels measured in shoot and seed samples are highly correlated, and processing of seed samples is faster and less expensive than shoot tissue (Barbosa et al., 2018).

Nodule traits (number and size), as an indicator of nitrogen fixing capacity, were not measured in this study. Numerous studies in dry bean have found that nodule traits are not correlated with nitrogen fixation capacity. For example, Farid (2015) found no correlation between nodule numbers and SNF, and in a study of SNF in the Middle American Diversity Panel (Wilker et al., unpublished) found no correlation between SNF and nodule size or nodule number. An in-field ureide assay was not feasible and a controlled environment study was not initiated for this panel.

To prepare for gas-chromatography mass-spectrometry (GCMS) analysis, a 5 g subsample of seed from each plot was oven-dried (Blue M Electric, SPX Corporation) at 60°C at the University of Guelph for 24 h prior to being ground to a coarse powder in a coffee grinder (various models used). The coarse seed powder was further processed into a fine powder suitable for gas chromatography mass spectrometry (GCMS) analysis by grinding a sub-sample in a small Eppendorf tube along with a steel bead in a bead mill (Beadruptor 12, Omni International Inc.). Samples (5 mg) of bean powder were measured into small tin capsules (8 mm × 5 mm, standard weight, Elemental Microanalysis) using an analytical balance (Quintix 65-1S, Sartorius Lab Instruments GmbH & Co.), enveloped and compressed into a tiny pellet so that no atmosphere remained in the capsule. The bean powder pellets were collected in 96-well plates and sent to the Agriculture and Agri-food Canada (AAFC) GCMS facility in Lethbridge, Alberta for analysis. The samples were analyzed with a Finnigan Delta V Plus (Thermo Electron, Bremen, Germany) Isotope Ratio Mass Spectrometer (IRMS) fitted with a Flash 2000 Elemental Analyzer (Thermo Fisher Scientific, Voltaweg, Netherlands) and Conflo IV (Thermo Fisher Scientific, Bremen, Germany) interface between the IRMS and the analyzer. A standardized curve for nitrogen content was created using an alfalfa standard provided by the AAFC GCMS facility. Further isotope standards L-glutamic acid USGS40 and USGS41 (United States Geological Survey) were included with each plate of samples processed to normalize isotope values and enable inter-lab comparison. Samples were analyzed for %N, δ^15^N (‰), and δ^13^C (‰).

The natural abundance method uses the following equation,

$$\begin{array}{c} \%Ndfa=\frac{\delta^{15}N\;reference\;plant-\delta^{15}N\;Nfixing\;plant}{\delta^{15}N\;reference\;plant\;-\;B} \end{array}$$

where, δ^15^N_ref.plant_ is the rate of δ^15^N in the reference genotype (R99), δ^15^N_fixingplant_ is the δ^15^N of the N-fixing bean genotype and B is the average δ^15^N of beans grown in an environment where its entire N source is from fixation (Peoples et al., 2009). The *B*-value was obtained for this experiment as described by Farid (2015). Briefly, δ^15^N was measured and averaged for 20 bean genotypes from both the Andean and Middle American genepools which were grown in a growth room in N-free media. Normalized δ^15^N values were used for all genotypes and an average of δ^15^N values for R99 were used in %Ndfa calculations.

### Genotyping

Leaf tissue samples were collected from young plants of 42 genotypes grown in a controlled environment (16 h photoperiod, 22°C) at the University of Guelph. For 29 genotypes, DNA was extracted using the manufacturer’s instructions for the NucleoSpin Plant II kit (Macherey-Nagel, Germany), and for the remaining 13 genotypes the DNeasy Plant Mini Kit (Qiagen, Canada) was used. DNA quality was tested using a spectrophotometer (ND-1000, Nanodrop) and a fluorometer (Qubit 2.0, Invitrogen by Life Technologies), and DNA of 39 genotypes was determined to be of sufficient quality to send for genotyping. Genomic DNA was analyzed at the Genome Quebec Innovation Centre (McGill University, Montreal, QC, Canada) for single nucleotide polymorphisms (SNPs) using the Illumina Infinium iSelect Custom Genotyping BeadChip (BARCBEAN6K_3) containing 5398 SNPs (Song et al., 2015).

### Identity by State Analysis

Single nucleotide polymorphism data from the above analysis was imported to TASSEL (Bradbury et al., 2007) for filtering such that the retained SNPs were present in 95% of the panel and the minor allele frequency was 0.05. This resulted in 39 genotypes and 4704 SNPs retained for further analysis. TASSEL was used to generate a genotype distance matrix and R software (R Core Team, 2013) was used to create a dendrogram using the *dendextend* package (Galili, 2015). The hierarchical clustering function, hclust (Müllner, 2013) was used to perform the cluster analysis using the UPGMA method. The as.dendrogram function was used to create dendrograms which were then modified in R using the *dendextend* package and the *circlize* package (Gu et al., 2014). STRUCTURE (Pritchard et al., 2000) was used to determine the population genetic structure of the HCP. The analysis was performed (20 replications) with the length of burn-in set at 5000 and the number of MCMC replications after burn-in set at 50000. A range of genetic groups (2K to 9K) were tested and the number that best fit the data was determined by visualizing the STRUCTURE results and using the ΔK statistic in STRUCTURE HARVESTER online (Evanno et al., 2005^8^; Earl and vonHoldt, 2012).

### Nucleotide Diversity Analysis

The levels of genetic diversity in the heirloom vs. conventional categories and the Andean vs. Middle American categories of the HCP were assessed. The π statistic provides an indication of polymorphism within a population as measured by nucleotide diversity (Nei and Li, 1979), and Tajima’s D provides an indication of selection pressure (Tajima, 1989). The 5K SNP dataset was used to calculate π and Tajima’s D with VCFtools 0.1.12b (Danecek et al., 2011), and MAF ≥ 0.01 and a window of 1000 bp was used. Genome-wide averages of π and Tajima’s D for each germplasm category were generated by taking the average across all windowed calculations. A *t*-test (GraphPad Prism8) was used to determine differences in both π and Tajima’s *D*-values between heirloom and conventional categories within each genepool.

### Statistical Analysis

Analysis of variance (ANOVA) tests were performed on the data collected from each environment and the environments combined using the MIXED procedure in SAS (version 9.4, SAS Institute, 2012. Cary, NC, United States). In each ANOVA, genotypes were considered fixed effects while all other effects and the interaction effects were considered random. The Shapiro–Wilks test (Shapiro and Wilk, 1965) was performed on the residuals in the UNIVARIATE procedure to test their normality. Random and independent distributions of the residuals were visually examined by plotting the studentized residuals against the predicted values. Data that generated outlier residuals were removed from the data set. Further, single degree of freedom contrasts were conducted in ANOVA between genotype categories, heirloom vs. conventional and Middle American vs. Andean. Repeated measures of leaf chlorophyll content (SPAD) were taken, and a separate ANOVA test was used to compare SPAD values at each time point. In each ANOVA, the genotype least squared means (LSmeans) were computed using the LSMEANS statement in the MIXED procedure.

The pair-wise Pearson’s coefficients of correlation were computed for all traits measured using the CORR procedure in SAS. The RINCOMP and PRINQUAL procedures were used in SAS to generate the principal component (PC) values, to estimate the proportion of variance accounted for by each PC, and to plot PC1 against PC2 to generate a genotype × trait (GT) biplot (Yan and Rajcan, 2002) to determine genotype and trait interactions overall and in each environment.

## Results

### Origins and Phenotypic Characteristics of Selected Beans

The germplasm comprising the HCP includes genotypes with a wide diversity of seed traits (colors, patterns, shapes, and sizes) found in dry bean. According to the descriptions from the source seed retailers, 16 of the heirloom genotypes are part of the cultural heritage of North American First Nations communities (the Algonquin, the Iroquois, the Seneca, and the Mohawk from the Great Lakes region of North America; the Arikara, the Hidatsa, and the Mandan from the Plains region in present-day United States). Genotype descriptions for the remaining nine heirloom genotypes suggest the varieties were passed down through communities or families from as far back as colonial times. For example, Sweeney Family Heirloom was first grown by the Sweeney family in Nova Scotia and has been moved with the family and grown in Alberta (Heritage Harvest Seeds). Further, while Sweeney Family Heirloom shows similarities to other heirloom genotypes, it is considered a unique variety by heirloom seed growers. Coco Sophie is a European variety from the 1700s (Heritage Harvest Seed). Amish Gnuttle (Amish Nuttle; also known as Cornhill Bean or Mayflower) is described by some retailers as a variety that was introduced to America with the early settlers and has been grown by Amish communities for generations, while other variety descriptions suggest that Amish Gnuttle originated with the Seneca First Nation.

The heirloom category was equally split between Andean and Middle American types (Table 1) and a variety of seed coat color patterns are represented, including bi-color, yellow eye, pinto/cranberry, and uncommon solid colors (Figure 1) which make them unique and difficult to categorize using conventional market classes. The conventional category was equally split between Andean and Middle American types and could mostly be categorized as kidney (dark red, light red, and white), cranberry, yellow, white, or black market class beans (Figure 1 and Table 1).

### Field Conditions

Fields with low nitrogen levels were used in this study to maximize the potential for SNF activity. In the growing seasons prior to 2014 and 2015, fields at the ERS had been planted with high-N demanding cereal crops to remove as much available nitrogen from the soil as possible. At the Belwood location, the field had been used to produce mixed hay with minimal inputs in the growing seasons previous to our trial. Soil test results showed that nitrate (NO_3-_) levels ranged between 3.7 and 8.6 ppm and ammonium (NH_4_) levels ranged between 2.6 and 6.1 ppm in the bean root zone. Soil analysis laboratory guidelines indicate that levels of NO_3-_ below 10 ppm are considered low (A & L Canada Laboratories Inc.).

Planting in 2015 occurred 2 weeks later than in 2014 as a result of wet spring weather. Despite the late start to the 2015 season, accumulated growing degree days (GDD) over the growing season were similar for all three locations (Elora 2014 – 1912.8, Elora 2015 – 1862.6, and Belwood 2015 – 2012.3). A summary of pre-plant soil test results, precipitation and total GDD for all location-years is provided in Supplementary Table 1.

### Genetic Analysis of Relatedness

**FIGURE 2:**
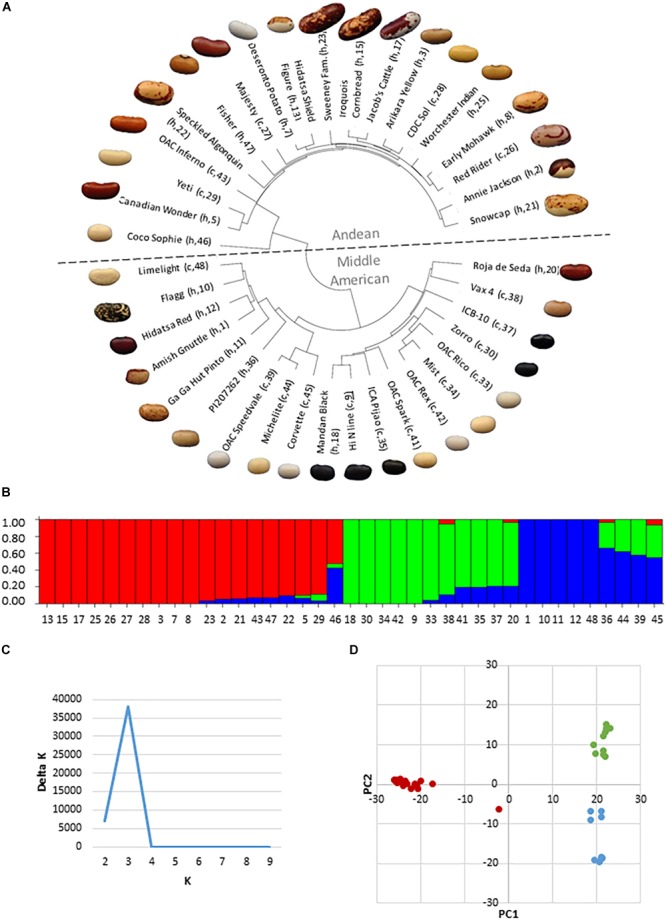
Analysis of genetic structure and relatedness of thirty-nine genotypes of the heirloom-conventional panel. **(A)** Dendrogram of genetic relatedness generated in R. Andean genotypes above and Middle American genotypes below the mid-line. Heirloom or conventional category membership is denoted by an “h” or “c,” respectively, along with the genotype code number; **(B)** STRUCTURE plot indicating the division of the panel into three genetic sub-groupings, Andean (red), Mesoamerica (green); and Durango-Jalisco (blue); **(C)** Delta K plot from fastSTRUCTURE indicating that the most appropriate sub-division of the panel is into three genetic groupings; **(D)** Principle component analysis plot confirming three genetic groupings in the panel.

The HCP was composed of genotypes from both the Middle American and Andean genepools, however the genepool composition and genetic relatedness of the genotypes was unknown. An identity-by-state (IBS) analysis on SNP genetic data from 39 genotypes of the HCP was undertaken to confirm genotype membership in either genepool and to determine the genetic relationships among them. The IBS analysis found that the panel is composed of three sub-groupings, with 19 genotypes belonging to the Andean genepool and 20 belonging to the Middle American genepool (11 race Mesoamerica and 9 race Durango-Jalisco). In the dendrogram (Figure 2A), large-seeded genotypes generally sorted into the Andean grouping while smaller-seeded genotypes sorted into the Middle American grouping. STRUCTURE analysis (Figure 2B) and determination of the best-fit ΔK value for the panel (Figure 2C) using STRUCTURE HARVESTER confirmed that there were three genetic groupings in the panel, corresponding to the Andean genepool and the two races (Mesoamerica and Durango-Jalisco) present in the Middle American genepool. The IBS analysis revealed the degree of genetic relatedness between modern and heirloom genotypes. For example, all of the black seed coat genotypes belong to the race Mesoamerica grouping of the Middle American genepool, and the University of Guelph breeding line, “Hi N” (Figure 2A,B, #9), is most closely related to the heirloom genotype Mandan Black (#18) and the conventional genotype ICA Pijao (#35), but it is less similar to Zorro (#30) and ICB-10 (#37). Assignment of varieties to either genepool based on genetic composition was generally in agreement with genepool assignments using seed characteristics, except for a few cases. For example, the large, flat-seeded Limelight (#48) and Flagg (#10) genotypes, which appear to be of Andean origin, belong by genetic analysis, to the Middle American genepool.

Evidence of admixture is apparent for a number of genotypes in the panel. Within the Middle American genepool, five of the genotypes are of entirely Durango-Jalisco and five are of entirely Mesoamerican ancestry. The remaining 10 Middle American genotypes are admixed between Durago-Jalisco and Mesoamerican races with 4 genotypes also containing <10% genetic material from the Andean genepool. Less admixture is evident within the Andean genepool, where 10 genotypes are entirely Andean and 8 genotypes contain <10% Middle American genetic material. Coco Sophie (Figure 2A,B, #46), a round, white bean of European heritage is unique in that it is approximately 50% Andean and 50% Middle American. In the principle component analysis (Figure 2D) Coco Sophie falls midway between the three genepool/race clusters. Repeated iterations of the STRUCTURE analysis of the panel assigned Coco Sophie to the Andean genepool 60% of the time, whereas on the basis of its seed color and shape this genotype would have been assigned to the Middle American genepool.

### Nucleotide Diversity Among Genotype Categories

Nucleotide diversity was measured in the HCP to ascertain whether genotypes comprising the heirloom category are more diverse than those in the conventional category, and similarly whether genotypes belonging to the Middle American genepool are more diverse than those belonging to the Andean genepool. According to the π and Tajima’s D statistics, nucleotide diversity for the heirloom category overall (π = 3.64 × 10^-4^, *D* = 7.262 × 10^-3^) was very similar to that found in the conventional category overall (π = 3.88 × 10^-4^, *D* = 7.908 × 10^-3^).

The number of SNPs among the Middle American genotypes in the HCP was 3294 compared to 2696 for the Andean genotypes. Nucleotide diversity using π, for the Middle American group (π = 3.64 × 10^-4^) was significantly (*p* = 0.0014) larger than for the Andean group (π = 2.13 × 10^-4^). Similarly, Tajima’s D statistic for the Middle American genepool (*D* = 0.79) was significantly higher (*p* = 0.0009) than for the Andean genepool (*D* = -0.18).

Nucleotide diversity between heirloom and conventional categories was further analyzed within the genepools. In the Middle American genepool, nucleotide diversity was not significantly different (π: *p* = 0.4137; D: *p* = 0.9783) between the heirloom (π = 4.08 × 10^-4^, *D* = 0.63) and the conventional genotypes (π = 3.61 × 10^-4^, *D* = 0.64). However, within the Andean genepool, heirloom nucleotide diversity was significantly higher (*p* = 0.0082) in conventional genotypes (π = 3.98 × 10^-4^) than heirloom genotypes (π = 2.35 × 10^-4^), but Tajima’s *D*-values were not significantly different (*p* = 0.1310) between heirloom (*D* = -0.09) and conventional genotypes (*D* = 0.47).

### Diversity for Seed Isotope Traits

**FIGURE 3:**
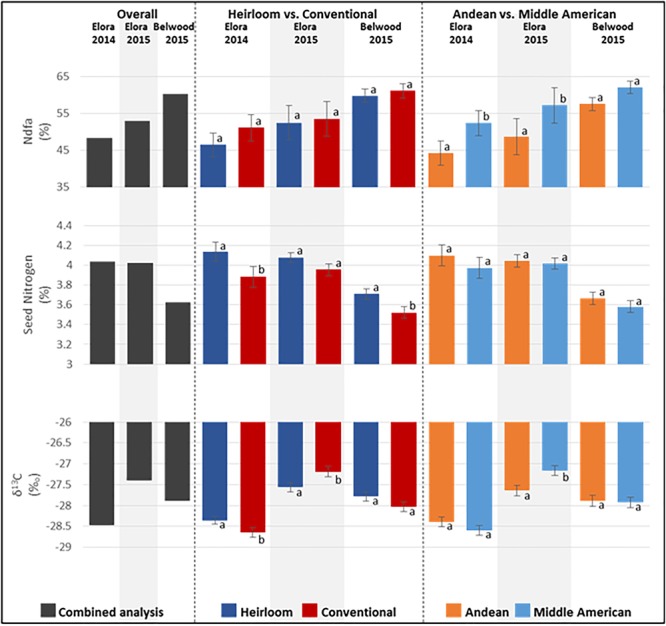
Means for seed composition traits measured from seed harvested at three field locations from genotypes of the heirloom-conventional panel. Comparisons within each year and subcategory ± standard error are presented. Means labeled with different letters within categories are significantly different according to ANOVA, *p* = 0.05.

Significant differences were seen among the genotypes for the seed traits analyzed by GCMS, including: nitrogen derived from the atmosphere (%Ndfa; *p* = 0.0002), seed nitrogen content (%N; *p* < 0.0001), and carbon discrimination (δ^13^C; *p* < 0.0001) (Figure 3 and Supplementary Table 2). Among the categories overall, significant differences were found for %Ndfa (*p* <0.0001), where Middle American genotypes (mean 62.16%) outperformed Andean (mean 54.82%) genotypes, and for seed nitrogen content (*p* < 0.0001), where heirloom genotypes (mean 3.97%N) contained higher levels of N than conventional (mean 3.79%N) genotypes. Significant differences were not found for other category comparisons of seed composition traits. While the effect of environment alone was not significant, the environment by genotype interaction effect (env^∗^ENTRY) was significant for all seed composition traits (Supplementary Table 2), and warranted further exploration.

When seed composition traits are analyzed for each location, significant genotype effects were found. At Elora 2014 (Figure 3 and Supplementary Table 3), significant differences were found between genotypes for %Ndfa (*p* = 0.0072), seed N content (*p* = 0.0105), and carbon discrimination (*p* = 0.0031). A comparison of genotype categories found significantly higher levels for %Ndfa (*p* = 0.0144) in Middle American genotypes (mean 54.37%) compared to Andean genotypes (mean 45.94%); and conventional genotypes (mean 53.06%) fixed more nitrogen than heirloom genotypes (mean 48.23%), although this difference was not statistically significant. For seed N content, significant differences (*p* = 0.0070) were seen at Elora 2014 where the heirloom category (mean 4.14%N) had higher seed N content than the conventional category (mean 3.88%N), however, no significant differences were seen between Andean (mean 4.1%N) and Middle American (mean 3.97%N) genotypes. For carbon discrimination (δ^13^C), significant differences (*p* = 0.0452) were found between heirloom (mean -27.5) and conventional (mean -27.8) genotypes, but not between Andean (mean -27.54) and Middle American (mean -27.75). Although significant differences were found among genotypes for %Ndfa (*p* = 0.0049), seed N content (*p* = 0.0126), and carbon discrimination (*p* = 0.0001) at Belwood in 2015 (Figure 3 and Supplementary Table 4), the only genotype category comparison where significant differences were found was for seed N content (*p* = 0.0251), where heirloom genotypes had higher %N (mean 3.71) than conventional genotypes (mean 3.52). At Elora 2015 (Figure 3 and Supplementary Table 5), significant differences were found between genotypes for %Ndfa (*p* = 0.0026), seed N content (*p* < 0.0001), and carbon discrimination (*p* = 0.0078), and comparisons of genotype categories found further significant differences. Similar to results for 2014, at Elora 2015 Middle American genotypes (mean 63.54%) fixed significantly (*p* = 0.0020) more nitrogen than the Andean genotypes (mean 54.19%), while the difference between heirloom (mean 58.36) and conventional (mean 59.59) was not significant (*p* = 0.6980). For seed N content at Elora 2015, no significant differences were seen between heirloom (mean 4.08%N) vs conventional (mean 3.95%N) or Andean (mean 4.04%N) vs Middle American (mean 4.02%N) categories. For carbon discrimination (δ^13^C), significant differences (*p* = 0.0233) were found between heirloom (mean -27.8) and conventional (mean -27.41) genotypes. Additionally, significant differences (*p* = 0.0049) between Andean (mean -27.9) and Middle American (mean -27.34) were found.

### Diversity for Agronomic Traits

**FIGURE 4:**
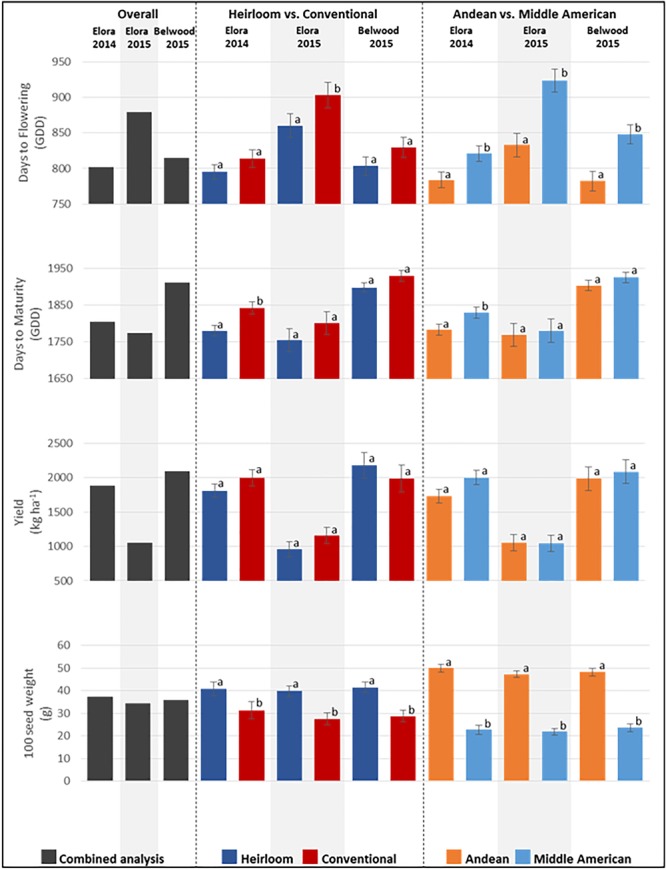
Means for agronomic traits measured at three field locations for the genotypes of the heirloom-conventional panel. Comparisons within each year and subcategory ± standard error are presented. Means labeled with different letters within categories are significantly different according to ANOVA, *p* = 0.05.

For agronomic traits, significant differences in the combined environments analysis were found among genotypes for days to flowering (GDD; *p* < 0.0001), days to maturity (GDD; *p* < 0.0001), yield (kg ha^-1^; *p* = 0.0003), and hundred seed weight (g; < 0.0001) (Figure 4 and Supplementary Table 2). Among categories overall, significant differences were found for days to flowering, where heirloom genotypes (mean 819.44 GDD) flowered significantly earlier than conventional genotypes (mean 849.83 GDD), and Andean genotypes (mean 798.80 GDD) flowered significantly earlier than Middle American genotypes (mean 865.44 GDD). Similarly, for days to maturity, heirloom genotypes reached maturity significantly earlier (mean 1811.24 GDD) than conventional genotypes (mean 1857.28 GDD). Significant differences were not found for either genotype category comparison for yield (kg ha^-1^), however, significant differences were found for 100 seed weight, where heirloom genotypes (mean 40.7 g) were larger than conventional genotypes (mean 28.8 g), and Andean (mean 48.35 g) genotypes were larger than Middle American genotypes (mean 22.70 g). While the effect of environment alone was not significant, the environment by genotype interaction effect (env^∗^ENTRY) was significant for days to flowering, yield and 100 seed weight (Supplementary Table 2), and warranted further exploration.

When agronomic traits are analyzed for each location, significant genotype effects were found. At Elora 2014 (Figure 4 and Supplementary Table 3), significant differences were found between genotypes for days to flowering (GDD; *p* = 0.0485), days to maturity (GDD; *p* < 0.0001), yield (kg ha^-1^; *p* = 0.0033), and 100 seed weight (g; *p* < 0.0001), and comparisons of genotype categories found further significant differences. For days to flowering, Middle American genotypes (mean 820.86 GDD) flowered significantly earlier than Andean genotypes (mean 783.60 GDD); and heirloom genotypes (mean 795.02 GDD) flowered earlier than conventional genotypes (mean 813.74 GDD), although this difference was not statistically significant. For days to maturity, heirloom genotypes (mean 1780.40 GDD) matured significantly earlier than conventional genotypes (mean 1842.20 GDD), and Andean genotypes (mean 1783.29 GDD) matured significantly earlier than Middle American genotypes (mean 1829.42 GDD). For yield, no significant differences were found between heirloom and conventional genotypes nor between Andean and Middle American genotypes. For 100 seed weight, heirloom genotypes had significantly higher weights (mean 40.82 g) than conventional genotypes (mean 31.35 g), and Andean genotypes (mean 49.97 g) were significantly heavier than Middle American genotypes (mean 22.69 g). At Belwood 2015 (Figure 4 and Supplementary Table 4), significant differences were found between genotypes for days to flowering (*p* < 0.0001), days to maturity (*p* < 0.0001), 100 seed weight (*p* < 0.0001). No significant differences were found among genotypes for yield. When category comparisons were performed, significant differences were found for days to flowering, with Andean genotypes (mean 782.02 GDD) flowering earlier than Middle American genotypes (mean 848.38 GDD). For 100 seed weight, heirloom genotypes (mean 41.51 g) were significantly heavier than conventional genotypes (mean 28.68 g), and Andean genotypes (mean 48.23 g) were significantly heavier than Middle American genotypes (mean 23.59 g). At Elora 2015 (Figure 4 and Supplementary Table 5), significant differences were found between genotypes for days to flowering (*p* =< 0.0001), days to maturity (*p* = 0.0002), yield (*p* < 0.0001), and comparisons of genotype categories found further significant differences for days to flowering and 100 seed weight. In particular, heirloom genotypes (mean 860.49 GDD) flowered significantly earlier than conventional genotypes (mean 903.76 GDD), and Andean genotypes (mean 833.02 GDD) flowered significantly earlier than Middle American genotypes (mean 924.12 GDD). For 100 seed weight, it was found that heirloom genotypes (mean 39.87 g) were significantly heavier than conventional genotypes (mean 27.53 g), and Andean genotypes (mean 47.24 g) were significantly heavier than Middle American genotypes (mean 21.80 g).

When random effects in the combined ANOVA are considered, the effect of environment is not significant for any trait, however, the genotype by environment interaction was significant for all traits, except Days to Maturity (Supplementary Table 2), indicating that genotype performance for most traits was affected by the growing environment. The block within environment interaction was not significant at any location, however, the incomplete block within the environment by block interaction was significant for %Ndfa, yield, and days to flowering (Supplementary Table 2), indicating some variation in performance across the field sites.

### Diversity for Leaf Chlorophyll Content

As a repeated measure, leaf chlorophyll content (SPAD) was analyzed in separate *F*-tests. Overall, SPAD values differed significantly by genotype during each field season (*p* < 0.0001, Supplementary Table 6) and at all locations, significant differences were found among genotypes for leaf chlorophyll content (*p* < 0.0001, Supplementary Table 6). In 2015, at both locations, significant differences were seen between SPAD measurements taken at different growth stages (early vegetative stage vs. reproductive stage) (Supplementary Table 6). Furthermore, at each location the growth stage at which leaf chlorophyll content was measured had a significant effect on genotype SPAD performance (significant SPADT^∗^G interaction; Supplementary Table 6). The observation within block by genotype by SPAD time interaction was significant in all environments (Supplementary Table 6).

Leaf chlorophyll content rating comparisons were also made between genotype categories using ANOVA. In 2014, no significant difference was found between heirloom and conventional genotypes (*p* = 0.7372), whereas Middle American genotypes had significantly higher SPAD ratings (mean SPAD value 37.19) than Andean genotype ratings (mean SPAD value 34.39). At Belwood 2015, significant differences (*p* = 0.0121) were found between heirloom (mean SPAD value 37.15) and conventional (mean SPAD value 38.90) genotypes, and further SPAD sampling time (*p* = 0.0002) and category^∗^SPADT interaction (*p* = 0.0164) were significant for the heirloom vs. conventional comparison. When genotypes were categorized according to genepool membership, significant differences (*p* = 0.0013) were found between Middle American (mean SPAD value 39.24) and Andean (mean SPAD value 36.43) genotypes. In addition, SPAD sampling time was significant (*p* = 0.0007), as was the interaction between genepool category and SPAD sampling time (*p* = 0.0222). At Elora 2015, no significant difference was found between heirloom and conventional genotypes (*p* = 0.7840), nor SPAD sampling time or the interaction (SPADT^∗^breeding category). When genotypes were compared according to genepool membership, significant differences (*p* < 0.0001) were found, where Middle American genotypes had significantly higher SPAD ratings (mean SPAD value 35.07) than Andean genotypes (mean SPAD value 31.86). Neither the SPADT nor the genepool^∗^SPADT interaction was significant at Elora in 2015.

### Nitrogen Fixation in the HCP

Table 2 ranks all genotypes in the panel for nitrogen fixing capacity as measured by %Ndfa. At Elora 2014, the %Ndfa range was between 20.8% (Jacob’s Cattle, heirloom, Andean) and 76.4% (Flagg, heirloom, Middle America) with an average value of 48.3%. At Elora 2015, the %Ndfa range was from 19.9% (Thermo Fisher Scientific, heirloom, Andean) to 70.9% (Coco Sophie, heirloom, Middle America) with an average value of 53.0%. At Belwood 2015, the %Ndfa range was from 43.5% (Limelight, conventional, Andean) to 76.3% (Hi N line, conventional, Middle American) with an average value of 60.3%.

**Table 2:** Nitrogen derived from the atmosphere (%) and differential ranking of common bean genotypes at three locations (Elora and Belwood) and in two seasons (2014 and 2015).

				Elora 2014		Elora 2015		Belwood 2015		Combined	
Code^#^	Genotype	Category	Genepool^‡^	%Ndfa	Rank	%Ndfa	Rank	%Ndfa	Rank	%Ndfa	Rank
46	Coco Sophie	Heirloom	Andean	NA	NA	70.9	1	75.7	3	69.0	1
9	Hi N line	Conventional	MA	67.2	2	61.2	8	76.3	1	66.9	2
18	Mandan black	Heirloom	MA	45.7	22	66.0	3	75.9	2	63.7	3
20	Roja de Seda	Heirloom	MA	60.4	6	54.0	21	75.0	4	62.7	4
36	PI207262	Heirloom	MA	51.2	15	60.0	12	74.7	5	62.4	5
10	Flagg	Heirloom	MA	76.4	1	59.4	14	61.8	18	62.4	6
38	Vax4	Conventional	MA	62.0	4	59.0	16	58.9	22	60.3	7
1	Amish Gnuttle	Heirloom	MA	63.4	3	66.3	2	48.8	37	59.8	8
43	OAC Inferno	Conventional	Andean	NA	NA	54.2	20	68.3	11	59.7	9
30	Zorro	Conventional	MA	45.1	24	59.2	15	69.5	8	59.3	10
11	Ga Ga Hut Pinto	Heirloom	MA	52.9	11	64.9	4	58.9	23	58.9	11
13	Hidatsa Shield Figure	Heirloom	Andean	53.1	10	59.7	13	63.0	15	58.7	12
23	Sweeney Family Heirloom	Heirloom	Andean	49.3	19	57.9	17	66.7	12	58.0	13
26	Red Rider	Conventional	Andean	60.6	5	52.5	22	62.1	16	57.4	14
37	ICB-10	Conventional	MA	42.4	27	61.7	6	64.6	14	57.1	15
42	OAC Rex	Conventional	MA	50.2	18	51.1	26	69.7	6	56.9	16
35	ICA Pijao	Conventional	MA	52.4	12	62.9	5	53.1	31	56.8	17
4	Canadian Wild Goose	Heirloom	MA	51.1	16	48.4	30	69.5	7	56.2	18
27	Majesty	Conventional	Andean	56.6	8	51.4	25	60.8	21	55.8	19
12	Hidatsa Red	Heirloom	MA	52.1	13	60.1	11	57.3	27	55.4	20
16	Kahnawake Mohawk	Heirloom	MA	48.2	20	48.3	31	69.2	9	55.2	21
34	Mist	Conventional	MA	35.2	30	61.2	7	68.7	10	54.5	22
44	Michelite	Conventional	MA	NA	NA	48.6	29	60.9	20	53.9	23
29	Yeti	Conventional	Andean	45.0	25	54.9	19	62.0	17	53.7	24
33	OAC Rico	Conventional	MA	45.2	23	51.8	24	58.2	25	53.3	25
15	Iroquois Cornbread	Heirloom	Andean	56.6	7	48.1	32	49.2	36	52.2	26
39	OAC Speedvale	Conventional	MA	36.4	29	60.2	10	61.4	19	52.1	27
5	Canadian Wonder	Heirloom	Andean	31.3	32	61.1	9	56.2	29	51.8	28
7	Deseronto Potato	Heirloom	Andean	51.1	17	48.7	28	57.8	26	50.9	29
28	CDC Sol	Conventional	Andean	55.4	9	37.7	38	56.8	28	50.3	30
41	OAC Spark	Conventional	MA	51.6	14	45.2	34	53.0	32	48.7	31
45	Corvette	Conventional	MA	NA	NA	56.3	18	50.2	35	48.4	32
21	Snowcap	Heirloom	Andean	47.8	21	44.6	35	46.2	39	48.1	33
22	Speckled Algonquin	Heirloom	Andean	29.9	33	44.3	36	66.0	13	47.6	34
2	Annie Jackson	Heirloom	Andean	35.0	31	52.3	23	58.7	24	47.6	35
25	Worchester Indian	Heirloom	Andean	39.8	28	49.0	27	50.7	34	46.6	36
3	Arikara Yellow	Heirloom	Andean	42.8	26	41.4	37	43.6	40	42.3	37
8	Early Mohawk	Heirloom	Andean	22.2	34	46.6	33	51.2	33	40.8	38
17	Jacob’s Cattle	Heirloom	Andean	20.8	35	37.0	39	54.1	30	38.3	39
48	Limelight	Conventional	Andean	NA	NA	35.8	40	43.5	41	35.8	40
47	Fisher	Heirloom	Andean	NA	NA	19.9	41	48.7	38	32.1	41
				**LSmean**	**Se**	**LSmean**	**Se**	**LSmean**	**Se**	**LSmean**	**Se**
	Heirloom			46.5	3.26	52.5	4.62	59.8	1.8	53.0	3.55
	Conventional			51.1	3.54	53.6	4.73	61.1	1.9	54.4	3.6
	Andean			44.3	3.34	48.6	4.9	57.6	1.8	50.3	3.45
	Middle American			52.4	3.35	57.1	4.86	62.1	1.7	57.2	3.44

*^#^Code for genotypes shown in Figure 1. ^‡^Genepool according to Gepts (1988) MA, Middle American.*

Although no differences were found in nitrogen fixing capacity between the heirloom and conventional genotype categories, when ranked overall, four of the top five genotypes for nitrogen fixation capacity in this study were heirloom genotypes (including: Coco Sophie, Mandan Black, Roja de Seda, and PI2017262). The conventional genotypes which ranked in the top ten for nitrogen fixation consist of two breeding lines (Hi N and Vax 4) and two recently released cultivars (OAC Inferno and Zorro).

In addition to desirable growth habit, the modern cultivars also possess disease resistance; the cream-colored Vax 4 is resistant to Common Bacterial Blight (CBB) and Bean Common Mosiac (BCM) virus (Singh et al., 2001), the light red kidney bean OAC Inferno is BCM and Anthracnose resistant (Smith et al., 2012), and the black bean Zorro is resistant to rust and Anthracnose and partially resistant to CBB (Kelly et al., 2009). Disease resistance and good nitrogen fixing performance make these genotypes desirable candidates for breeding programs. Nitrogen fixing capacity was consistently higher in Middle American than Andean genotypes, and four of the top five nitrogen fixing genotypes belong to the Middle American genepool (Mandan Black, Roja de Seda, PI207262, and Hi N line).

### Trait Correlation

At Elora 2014, the correlation between days to flowering and days to maturity and the correlation between the first and second SPAD measurement time were positive and significant (Supplementary Table 7). At Elora 2015, significant, positive correlations were found between %Ndfa and all traits except yield; a significant, negative correlation was seen that year between seed N and yield (Supplementary Table 8). Similarly, at Belwood 2015, significant, positive correlations were seen for %Ndfa and all traits except yield and δ^13^C (Supplementary Table 9). Yield was not found to be significantly correlated with any trait in 2015 at either location (Supplementary Tables 8, 9).

**FIGURE 5:**
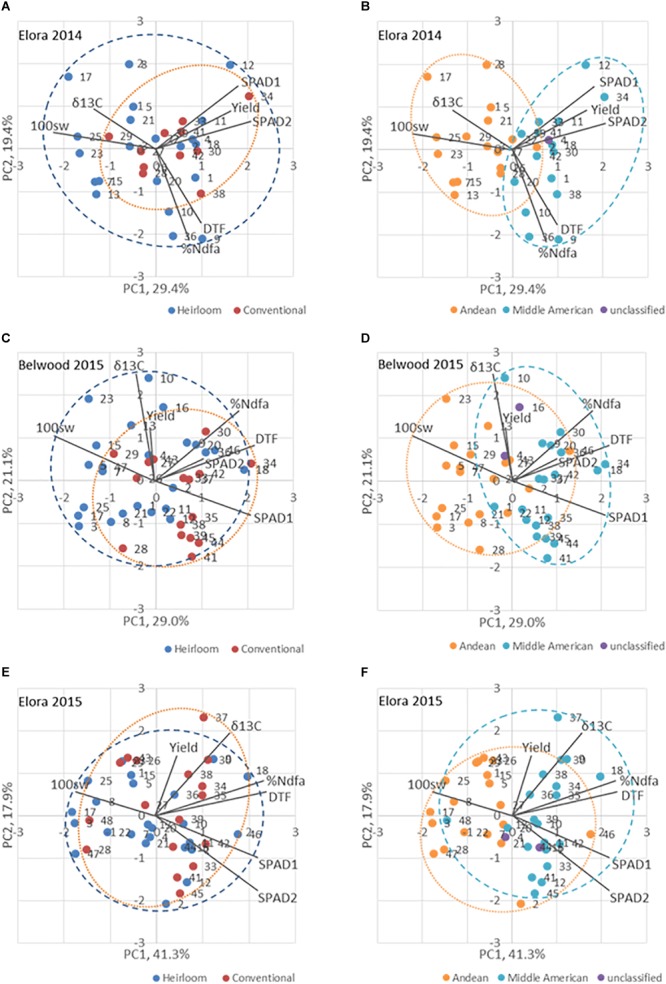
Biplot analysis of traits for genotypes of the heirloom-conventional panel in three location-years. Panels **(A,C,E)** divide the panel based on Heirloom/Conventional-bred categories. Panels **(B,D,F)** divide the panel based on Andean and Middle American genepool membership. The ellipses encompass all the genotypes of a particular category. DTF, days to flowering; Yield, yield (kg ha^-1^); 100sw, hundred seed weight (g); δ13C, carbon discrimination; %Ndfa, percent nitrogen derived from the atmosphere; SPAD1, leaf chlorophyll content, second trifoliate stage; SPAD 2, leaf chlorophyll content, 100% flowering.

The first two principle components in trait biplots (Figure 5) accounted for 49.9% of the variation in Elora 2014 (Figure 5A,B), 64.9% in Elora 2015 (Figure 5C,D), and 51.3% in Belwood 2015 (Figure 5E,F). The positive relationships between days to flowering and %Ndfa at each location-year are indicated by the acute angle formed by the vectors for these traits. The near-right angles formed by the %Ndfa and SPAD vectors at each location-year indicate that no relationship exists between these traits. The obtuse angle formed by the carbon discrimination (δ^13^C) and %Ndfa vectors in Elora 2014 indicates a negative relationship between these traits, while in 2015 the vectors are closer together forming a smaller angle and indicating a closer relationship.

When genotypes are categorized according to breeding history (Figure 5A,C,E), the conventional and heirloom genotypes occupy largely overlapping areas of the plot. However, when the genotypes are categorized according to genepool membership (Andean vs Middle America, Figure 5B,D,F), a significant fraction of the Andean population falls exclusively into areas defined by PC1. In these representations the Middle American genotypes are clustered in the direction of the %Ndfa vector.

## Discussion

### Genetic Diversity Is Greater in the Middle American Than the Andean Genepool

The IBS and nucleotide diversity analyses of the HCP was in accordance with the often-observed higher level of genetic diversity within the Middle American genepool compared to the Andean genepool. Multiple studies have found higher levels of diversity in the Middle American genepool than the Andean (Koenig and Gepts, 1989; Beebe et al., 2000, 2001; Papa and Gepts, 2003; McClean et al., 2004; Mamidi et al., 2011, 2013; Bellucci et al., 2014; Schmutz et al., 2014). In a study of AFLP and SSR marker diversity in domesticated and wild bean populations, Rossi et al. (2009) found evidence of a bottleneck event before domestication in the Andean genepool. Bitocchi et al. (2012) also found significant differences in genetic diversity between wild Middle American and Andean genotypes lending support to the occurrence of a genetic bottleneck prior to domestication of the Andean genepool. Therefore, the current low level of diversity among domesticated Andean genotypes was caused by bottlenecks during the establishment of the wild progenitor bean populations and during domestication (Bitocchi et al., 2013). The HCP has similar nucleotide diversity and no genetic differentiation between heirloom and conventional genotypes.

Decades of breeding, based on the use of a limited pool of elite cultivars has generated concern that this practice has led to a narrowing of crop genetic diversity in modern varieties (Plucknett et al., 1987; Gepts, 2006). However, the perception that heirloom genotypes are more genetically diverse than varieties from modern breeding programs was not supported by the genetic diversity analysis of the HCP. The interspersion of heirloom and conventional genotypes around the dendrogram (Figure 2) suggests that decades of isolated development of these two germplasm categories has not led to genetic divergence. Furthermore, genetic diversity measurements with the π and Tajima’s D statistics were not significantly lower for conventional genotypes than heirloom genotypes in this study. This was true for the overall comparison and the comparison within the Middle American genepool. Within the Andean genepool, greater nucleotide diversity was indicated by π and Tajima’s D within the conventional genotypes compared to the heirloom genotypes.

While this finding is in accordance with analyses performed in other crop species which concluded that modern breeding practices have not reduced genetic diversity (van de Wouw et al., 2010), it contradicts a recent comprehensive study in bean based on SSR marker diversity among wild, landrace and modern American genotypes of each genepool that concluded that genetic diversity has been lost as a result of breeding practices (Gioia et al., 2019). The contradictory conclusions may be related to differences in the marker systems and number of markers that were used in the studies; 24 SSR markers were used in the Gioia et al. (2019) study versus more than 4700 SNP markers in the current study. In addition, the number of individuals that were analyzed differed, with 192 advanced bean cultivars plus 349 accessions of wild plus domesticated beans used in the Gioia et al. (2019) study versus 25 heirloom and 17 conventionally bred dry bean genotypes in the present study. However, it is likely the case that the difference is related to the fact that both the heirloom and conventional varieties used in the present study were selected materials that have both been subjected to a domestication bottleneck. Our results suggest that modern practices have not introduced another significant loss in genetic diversity.

### Nitrogen Fixation Capacity in Middle American Genepool Exceeds That Found in Andean Genepool

Although the range for nitrogen fixation among genotypes in the Middle American genepool (Mist, 35.2%Ndfa to Hi N, 76.3%Ndfa) was narrower than in the Andean genepool (Fisher, 19.9%Ndfa to Coco Sophie, 75.7%Ndfa), nitrogen fixation among Middle American genotypes (average = 62.2%Ndfa) was significantly higher than among the Andean genotypes (average = 54.8%Ndfa). This suggests that the genes controlling nitrogen fixation capacity may differ between the genepools, perhaps both in number of loci and their diversity. However, few studies exist that compare the nitrogen fixing capacities of Middle American genotypes with Andean genotypes. Ramaekers (2011) identified a few quantitative trait loci (QTL) associated with SNF-capacity using a recombinant inbred line (RIL) population created from a cross between an Andean and a Middle American genotype. Other studies have used sets of either Middle American or Andean genotypes. For example, Kamfwa et al. (2015) studied 259 genotypes belonging to the Andean Diversity Panel (Cichy et al., 2015), and a 188 F_4:5_ RIL population derived from two Andean parents (Kamfwa et al., 2019) and found a number of QTL associated with nitrogen fixation. Similar studies with Middle American germplasm have identified similar as well as unique QTL associated with nitrogen fixation (Farid, 2015; Diaz et al., 2017; Heilig et al., 2017; Wilker and Pauls, 2019). Further research to identify QTL associated with nitrogen fixation in a panel comprised of genotypes from each genepool followed by assessment of haplotype diversity at the QTL would provide information on whether Middle American genotypes contain a greater number of active sites for N fixation than Andean genotypes or unique, more effective alleles. The higher levels of SNF in the Middle American genepool may be attributable to the higher level of genetic diversity on the Middle American genepool overall, as confirmed in this study. Alternatively, the Middle American genotypes may have performed better with the Rhizobia inoculant and/or strains present in the soil.

### Diversity for Nitrogen Fixation in Conventional Bean Genotypes Similar to Other Studies

Nitrogen fixation (%Ndfa) among the 18 conventional genotypes in the HCP (excluding R99) ranged from the lowest overall ranked AAFC-bred Limelight historic variety at 35.8% to the highest overall ranked University of Guelph breeding line Hi N at 66.9% (Table 2). These results fall generally within the range of %Ndfa reported for beans in contemporary research studies using conventional genotypes but other studies of nitrogen fixation, using conventional genotypes, have reported a broader range for this trait. For example Kamfwa et al. (2015) found a range from 3.6 to 98.2%Ndfa in their study of the 259-genotype Andean Diversity Panel and a study with 79 Middle American genotypes under organic production (Heilig et al., 2017) reported a range of 9.8 to 71.1%Ndfa. Early studies of nitrogen fixation in bean (Graham and Rosas, 1977; Graham, 1981) reported that fixation varied according to plant architecture, where determinate bush types had poorer performance than indeterminate climbing types. Economically viable seed yields (1000–2000 kg ha^-1^) were not attainable when plant %Ndfa levels were low, although variation for nitrogen fixation was acknowledged (Bliss, 1993). Therefore, the 18 conventional genotypes in the HCP, spanning decades of cultivar releases by breeding programs across North America, likely represent the mid-range of nitrogen fixing capacity among conventional bean genotypes.

### Modern Breeding Has Not Reduced SNF Capacity

This study showed that despite decades of modern production and breeding practices, which include the use of nitrogen fertilizer that downregulates SNF activity, SNF capacity has not been lost from conventional genotypes. Recently released varieties such as Zorro, a black bean developed at Michigan State University (Kelly et al., 2009), and OAC Inferno, a light red kidney bean developed at the University of Guelph (Smith et al., 2012), showed good performance for nitrogen fixation in our study. OAC Inferno also performed well in a study examining SNF in the Andean Diversity Panel in Michigan (Kamfwa et al., 2015). The breeding methodologies used to develop Zorro and OAC Inferno are representative of modern breeding practices. Zorro was developed by pedigree and pure line selection from a backcross population generated from a bi-parental cross of Michigan State University black bean breeding lines (B00103 and X00822), with emphasis on selection for disease resistance, plant architecture and yield. OAC Inferno was derived from a conical cross of diverse kidney bean variety parentage (HR85-1885/Montcalm//USWA-39/AC Litekid///Foxfire/AC Elk//Sacramento/AC Calmont) sourced from across North America, using disease resistance and yield as selection criteria. Kamfwa et al. (2015) found that OAC Inferno was the only genotype in that study to contain major effect alleles for Ndfa at three loci. The complex pedigree of OAC Inferno may have contributed to its genetic diversity and higher than usual capacity for nitrogen fixation in this Andean genotype.

The finding that SNF in the heirloom category overall was not superior to the conventional category did not support the hypothesis on which the study was based and may be attributable to the composition of the HCP. The panel is small and was designed to include a broad representation of bean genotypes; the heirloom cultivars come from wide geographic origins and are of unspecified breeding heritage (landraces and vintage varieties), and the modern genotypes include those released across recent decades as well as recent, elite modern cultivars. Different results may have been achieved had the study included wild bean germplasm and landraces and more-recently registered modern cultivars.

### Incorporating Heirloom Genotypes Into Breeding for Improved SNF Holds Potential

Previous to this study, there was no indication that nitrogen fixation capacity would be superior in heirloom bean genotypes. The discovery of the diversity in capacity for nitrogen fixation among the 23 heirloom genotypes in the HCP [ranging from the lowest overall ranked genotype (Fisher at 32.1%) to the highest overall ranked (Coco Sophie at 69.0%, Table 2)] suggests that heirloom varieties may be an excellent germplasm resource for studying this trait. Furthermore, we found a wide range in capacity for nitrogen fixation and yield performance among the heirloom genotypes of the HCP that was *on par* with conventional genotypes, indicating the suitability of heirloom beans for incorporation into breeding programs. In addition, the ranked panel for SNF performance (%Ndfa), was dominated by heirloom genotypes. Heirloom bean landraces are not routinely used to breed conventional varieties. For example, Navabi et al. (2014) undertook a pedigree analysis of Canadian dry bean varieties since the 1930s, and while a few introgressions of *P. coccinius* and *P. acutifolius* were made, heirloom genotypes were not evident, except among the oldest crosses. Heirloom beans possess diversity that could be exploited without the challenges encountered when breeding with wild relatives, such as infertile crosses and reintroduction of ‘wild’ traits. In addition, heirloom varieties grown by First Nations groups for centuries in the Great Lakes region of North America, are well-adapted to the climate and soils and perhaps the Rhizobium of this region.

Additionally, Coco Sophie (#46), which is unique in the HCP for its admixture between the genepools and is representative of European bean germplasm (Gioia et al., 2013), might be used as a bridge parent to transfer desirable traits from one genepool to the other (Duc et al., 2015). In particular, because Coco Sophie already possesses good SNF capacity it could be useful to introgress SNF traits from higher-fixing Middle American germplasm to lower-fixing Andean germplasm.

The similar yield of heirloom and conventional categories indicates heirloom genotypes have breeding potential in modern programs. When genotypes of the HCP were compared based on breeding history, no significant difference was found in yield between heirloom (1651 kg ha^-1^) and conventional (1714 kg ha^-1^) groups. A number of explanations for the similar yield performance of heirloom and conventional genotypes in the present study are plausible. Firstly, heirloom varieties were sourced from commercial seed suppliers and the HCP may have been enriched in heirloom lines that had reasonable performance characteristics. Secondly, low soil nitrogen levels may have limited the yield performance of conventional genotypes, which have been bred to perform under intensive management regimes. And finally, the conventional genotypes were not chosen for the panel based on superior yield potential but on market class similarity to heirloom genotypes in the panel. Some of the conventional genotypes were registered as long ago as the 1940s, and yields in bean crops grown in Ontario have increased by 1000 kg ha^-1^ in three decades (OMAFRA, 2016) and comparisons of bean varieties released over 40 years produced under conventional conditions show that breeding has increased their yield potential by more than 1% per year (Navabi et al., personal communication). Overall, the yield performance of the heirloom genotypes in our study would suggest that incorporation of these genotypes into a modern breeding program for organic production would not introduce significant yield drag. Singh et al. (2011) suggested that the use of well-adapted heirloom genotypes in bean breeding could be “crucial for developing high-yielding broadly adapted cultivars for sustainable organic and conventional production systems, thus reducing research and production costs.”

### Heirloom Beans May Be Particularly Suited to Breeding for Organic Agriculture

The rise in demand for organic food has broadened societal interest in heirloom varieties. Heirloom genotypes may be inherently well suited to organic production practices where growing conditions share similarities with the environments in which First Nations peoples grew them (Singh et al., 2011). Heirloom beans often possess characteristics such as attractive seed coat colors and patterns, desirable texture and flavor, and heritage value which increase their marketability and make them attractive to organic growers (Boyhan and Stone, 2016). Culinary characteristics were found to be of particular importance to heirloom bean growers in one study (Brouwer et al., 2016), while unique seed coat patterns as well as flavor and texture characteristics were emphasized by growers in another study (Swegarden et al., 2016).

Conventional varieties lack traits which give them a competitive advantage in low-input productions systems and may hamper their yield performance. However, modern, conventionally bred crop varieties account for more than 95% of varieties grown in organic production (Lammerts van Bueren et al., 2011). Direct comparisons of the yield performance of heirloom and conventional genotypes under organic production show mixed results. Miles et al. (2015) found that yield did not differ significantly between heirloom (1852 kg ha^-1^) and conventional (1983 kg ha^-1^) groups, whereas, Swegarden et al. (2016) found that heirloom genotypes (1362 kg ha^-1^) yielded significantly less than the conventional genotypes (2447 kg ha^-1^). In an evaluation of a large panel of conventional black and navy bean genotypes under organic production the yields ranged from 1228 to 1762 kg ha^-1^ (Heilig et al., 2017), which is similar to the range found in the current study (1160–2002 kg ha^-1^) of heirloom and conventional genotypes under low nitrogen management.

In the present study, weed growth was difficult to manage, and lesions symptomatic of Common Bacterial Blight or Anthracnose were found on various genotypes (disease notes not recorded). Therefore, the development of genotypes exhibiting early canopy closure and disease resistance might be particularly advantageous for organic production systems. Studies in bean comparing the outcome of selection under organic and conventional growth conditions resulted in different genotypes being chosen based on yield performance (Singh et al., 2011). Similarly in soybean, Boyle (2016) found that selection performed under organic production favored genotypes with improved performance for resource acquisition traits (early canopy development, nodule mass, and root length).

## Conclusion

This study represents the first comparison of SNF in a panel of heirloom and conventional dry beans and will serve as a starting point for further research on promising heirloom genotypes. The finding that genetic diversity is similar between heirloom and conventional categories is consistent with the finding that %Ndfa in heirloom and conventional categories is not significantly different. This result does not support the hypothesis that genetic diversity for nitrogen fixation has been eroded over years of modern breeding practices. The heirloom genotypes, as a group, had similar yield performance to the conventional genotypes under low-input field conditions, and although their capacity for nitrogen fixation was not significantly better than the conventional genotypes, they dominate the list of the best nitrogen fixers. Considering these characteristics, heirloom genotypes hold some promise for breeding to improve nitrogen fixation capacity in modern bean varieties. Heirloom beans represent an underutilized resource which could be exploited to improve nitrogen fixation in breeding for organic production and conventional production where reduction of synthetic inputs and improved environmental stewardship are of growing concern.

## Data Availability

Publicly available datasets were analyzed in this study. This data can be found here: https://doi.org/10.5683/SP2/NZY3W5.

## Author Contributions

JW, AN, and KP designed the project. JW performed the experiments and carried out the seed analysis. BH and FM helped in the seed analysis experiments. DT carried out the genetic diversity analysis. JW and AN analyzed the data. AN, KP, and IR guided the experimental work. JW and KP wrote the manuscript.

## Conflict of Interest Statement

The authors declare that the research was conducted in the absence of any commercial or financial relationships that could be construed as a potential conflict of interest.

## Footnotes

1 http://www.heritageharvestseed.com

2 http://www.assiniboinetipis.com

3 http://www.annapolisseeds.com

4 https://doi.org/10.5683/SP2/NZY3W5

5 https://www.uoguelph.ca/ses/service/weather-records

6 http://climate.weather.gc.ca/index_e.html

7 https://www.specmeters.com/assets/1/22/2900P_SPAD_502.pdf

8 http://taylor0.biology.ucla.edu/structureHarvester/
