# Supplementary material for: Agronomic Performance and Nitrogen Fixation of Heirloom and Conventional Dry Bean Varieties Under Low-Nitrogen Field Conditions
Source: Front Plant Sci. 2019 Jul 26;10:952. doi: 10.3389/fpls.2019.00952 (PMC6676800; doi:10.3389/fpls.2019.00952)
Supplement: Supplementary file 1 [file Data_Sheet_1.PDF]

Supplementary Table 1. Description of trial sites of the 2014-15 low-nitrogen dry bean (*Phaseolus vulgaris* L.) field trials.

		Elora 2014		Elora 2015		Belwood 2015	
Soil test (ppm)[†]		NO_{3-}	NH_3	NO_{3-}	NH_3	NO_{3-}	NH_3
	0-15cm	5.7	3.4	5.9	6.1	6.4	3.5
	15-30cm	8.6	2.9	3.7	3.0	5.5	2.6
Planting date		14-Jun		26-Jun		25-Jun	
First killing frost date		11-Oct		17-Oct		17-Oct	
Total growing days		124		114		115	
Total growing degree days (GDD)[‡]		1912.8		1862.6		2012.3	
Precipitation (mm)[§]							
	June	68.9		204.7		198.3	
	July	133.7		68.6		41.5	
	August	51.0		104.8		70.2	
	September	164.8		47.9		26.8	
	October	74.3		108.2		117.3	

[†] Soil tests performed by SGS Agrifood Laboratories (Guelph) on composite samples across the field sites of the top layer (0-15 cm) and mid layer (15-30 cm) of soil. Nitrate nitrogen (NO_{3-}) and Ammonium nitrogen (NH_3) are reported in parts per million (ppm).

[‡] Growing degree days (GDD, base '0') were calculated based on the total growing days for each location-year.

[§] Rainfall and temperature data for the Elora Research Station (Elora) were collected by the School of Environmental Sciences; rainfall and temperature data for the Belwood field site are as reported from the nearest Canada Weather Service location at the Fergus Shand Dam.

Supplementary Table 2. F-test of fixed effect of genotype, and variance component estimates (S^2) and their standard error (Se) of random effects in the combined mixed-model analysis of 41 heirloom and modern genotypes tested in multiple field locations in Ontario, Canada, 2014-2015.

	Ndfa§ (%)		Seed N content (%)		$\delta^{13}C$◊ (‰)		Flowering (GDD)		Maturity (GDD)		Yield ($kg\ ha^{-1}$)		Hundred seed weight (g)	
Fixed effect†	F-test	P-value	F-test	P-value	F-test	P-value	F-test	P-value	F-test	P-value	F-test	P-value	F-test	P-value
Genotype	2.61	0.0002	3.0200	<.0001	2.82	<.0001	11.47	<.0001	18.50	<.0001	2.52	0.0003	129.23	<.0001
H vs. C	2.01	0.1576	15.8900	<.0001	0.04	0.8362	8.37	0.0043	14.42	0.0002	0.55	0.4594	34.86	<.0001
A vs. M	19.54	<.0001	2.5500	0.1122	1.51	0.2211	44.01	<.0001	4.36	0.0385	1.95	0.1644	385.8	<.0001
Random effects‡	S^2	Se	S^2	Se	S^2	Se	S^2	Se	S^2	Se	S^2	Se	S^2	Se
env	31.06^{ns}	42.388	0.05432^{ns}	0.057	0.1028^{ns}	0.108	1796.49^{ns}	1914.220	3878.29^{ns}	4058.160	0.2828^{ns}	0.297	0.1571^{ns}	0.6633
BLOC(env)	14.64^{ns}	16.086	0.002709^{ns}	0.004	0.00	.	166.43^{ns}	162.100	279.56^{ns}	293.980	0.01150^{ns}	0.020	0.389^{ns}	0.6545
IBLK(env*BLOC)	22.51*	9.865	0.000	.	0.00	.	0^{ns}	.	185.1^{ns}	140.520	0.05259*	0.027	1.5325*	0.8413
env*ENTRY	25.56*	11.315	0.01879*	0.010	0.08428*	0.039	592.93**	220.630	129.03^{ns}	195.370	0.05933*	0.035	1.6721*	1.0016
Residual	64.55***	10.053	0.0703***	0.010	0.2513***	0.034	1285.23***	171.160	1349.99***	227.500	0.2181***	0.034	6.0486***	0.9841

† Genotype effects overall and by subcategory, heirloom (H) vs. conventional (S), and Andean (A) vs. Middle American (M) genepool

‡ Environment (env), block (BLOC), and incomplete block (IBLK)

§ Nitrogen derived from the atmosphere

◊ Carbon discrimination, normalized

^{ns} Not significant; *, **, and *** are significant at 0.05, 0.01, and 0.001, respectively

Supplementary Table 3. F-test of fixed effect of genotype, and variance component estimates (S^2) and their standard error (Se) of random effects in the combined mixed-model analysis of 36 heirloom and modern genotypes tested at Elora, Ontario, Canada, 2014.

	Ndfa§ (%)		Seed N content (%)		$\delta^{13}C$◊ (‰)		Flowering (GDD)		Maturity (GDD)		Yield (kg ha^{-1})		Hundred seed weight (g)	
Fixed effect†	F-test	P-value	F-test	P-value	F-test	P-value	F-test	P-value	F-test	P-value	F-test	P-value	F-test	P-value
Genotype	3.26	0.0072	2.9400	0.0105	3.66	0.0031	2.00	0.0485	9.38	<.0001	3.48	0.0033	49.19	<.0001
H vs. C	2.23	0.1420	7.6700	0.0079	4.22	0.0452	1.67	0.2015	11.36	0.0014	1.54	0.2196	4.86	0.0340
A vs. M	6.47	0.0144	1.7000	0.1983	2.03	0.1613	6.32	0.015	5.54	0.0227	3.28	0.0764	106.83	<.0001
Random effects‡	S^2	Se	S2	Se	S^2	Se	S^2	Se	S^2	Se	S^2	Se	S^2	Se
BLOC	17.0987^{ns}	27.802	0.007079^{ns}	0.019	0.000	.	0.000	.	0.00	.	0	.	0	.
IBLK(BLOC)	0.000	.	0.02754^{ns}	0.026	0.06848^{ns}	0.05	0.000	.	132.47^{ns}	249.070	0	.	7.3088^{ns}	6.7337
Residual	74.09***	19.801	0.06497**	0.022	0.1178**	0.04	2458.65***	596.310	1149.42***	357.660	161564***	41037	6.0957^{ns}	3.8924

† Genotype effects overall and by subcategory, heirloom (H) vs. conventional (S), and Andean (A) vs. Middle American (M) genepool

‡ Block (BLOC), and incomplete block (IBLK)

§ Nitrogen derived from the atmosphere

◊ Carbon discrimination, normalized

^{ns} Not significant; *, **, and *** are significant at 0.05, 0.01, and 0.001, respectively

Supplementary Table 4. F-test of fixed effect of genotype, and variance component estimates (S^2) and their standard error (Se) of random effects in the combined mixed-model analysis of 41 heirloom and modern genotypes tested at Belwood, Ontario, Canada, 2015.

	Ndfa§ (%)		Seed N content (%)		$\delta^{13}C$◊ (‰)		Flowering (GDD)		Maturity (GDD)		Yield (kg ha^{-1})		Hundred seed weight (g)	
Fixed effect†	F-test	P-value	F-test	P-value	F-test	P-value	F-test	P-value	F-test	P-value	F-test	P-value	F-test	P-value
Genotype	2.64	0.0049	2.3000	0.0126	4.19	0.0001	17.15	<.0001	7.64	<.0001	1.52	0.1296	68.24	<.0001
H vs. C	0.31	0.5779	5.2500	0.0251	2.96	0.0899	1.95	0.1672	2.50	0.1193	1.27	0.2639	15.14	0.0002
A vs. M	3.68	0.0596	0.9900	0.3228	0.06	0.8043	14.08	0.0000	1.24	0.2699	0.41	0.5224	113.99	<.0001
Random effects‡	S^2	Se	S2	Se	S^2	Se	S^2	Se	S^2	Se	S^2	Se	S^2	Se
BLOC	0.000	.	0.00	.	0.01^{ns}	0.025	179.340^{ns}	279.910	0	.	27905^{ns}	84308	1.3469^{ns}	2.3289
IBLK(BLOC)	0.000	.	0.00	.	0.00	.	0.000	.	0	.	147548^{ns}	115378	0.82^{ns}	1.5484
Residual	63.3036***	14.155	0.08644***	0.019	0.1683***	0.038	771.6***	172.530	1631.7***	390.05	373670***	100159	7.0451***	1.9072

† Genotype effects overall and by subcategory, heirloom (H) vs. conventional (S), and Andean (A) vs. Middle American (M) genepool

‡ Block (BLOC), and incomplete block (IBLK)

§ Nitrogen derived from the atmosphere

◊ Carbon discrimination, normalized

^{ns} Not significant; *, **, and *** are significant at 0.05, 0.01, and 0.001, respectively

Supplementary Table 5. F-test of fixed effect of genotype, and variance component estimates (S^2) and their standard error (Se) of random effects in the combined mixed-model analysis of 41 heirloom and modern genotypes tested at Elora, Ontario, Canada, 2015.

	Ndfa§ (%)		Seed N content (%)		$\delta^{13}C$◊ (‰)		Flowering (GDD)		Maturity (GDD)		Yield (kg ha^{-1})		Hundred seed weight (g)	
Fixed effect†	F-test	P-value	F-test	P-value	F-test	P-value	F-test	P-value	F-test	P-value	F-test	P-value	F-test	P-value
Genotype	2.91	0.0026	4.7000	<.0001	2.44	0.0078	19.4300	<.0001	11.2500	0.0002	5.45	<.0001	99.55	<.0001
H vs. C	0.15	0.6980	3.3200	0.0731	5.39	0.0233	4.780	0.0320	3.6100	0.0640	3.0700	0.0843	15.17	0.0002
A vs. M	10.45	0.0020	0.1300	0.7213	8.53	0.0049	24.920	<.0001	0.1700	0.6825	0.0100	0.9437	169.89	<.0001
Random effects‡	S^2	Se	S2	Se	S^2	Se	S^2	Se	S^2	Se	S^2	Se	S^2	Se
BLOC	32.3229^{ns}	60.210	0.000	.	0.00	.	329.6^{ns}	500.1100	1253.11^{ns}	1974.85	0	.	0	.
IBLK(BLOC)	60.3893*	34.019	0.01057^{ns}	0.008	0.00	.	32.1592^{ns}	123.9400	606.39^{ns}	542.7	57235^{ns}	32589	0.234^{ns}	0.7947
Residual	59.968***	16.583	0.02858***	0.008	0.3193***	0.07	773***	201.770	1072.99**	431.76	70697^{ns}	18948	4.643***	1.246

† Genotype effects overall and by subcategory, heirloom (H) vs. conventional (S), and Andean (A) vs. Middle American (M) genepool

‡ Block (BLOC), and incomplete block (IBLK)

§ Nitrogen derived from the atmosphere

◊ Carbon discrimination, normalized

^{ns} Not significant; *, **, and *** are significant at 0.05, 0.01, and 0.001, respectively

Supplementary Table 6. F-test of fixed effects and random effects in the mixed-model analysis of leaf chlorophyll content (SPAD) for 48 heirloom and modern genotypes tested at three field locations in Ontario, Canada, 2014-2015.

	Elora 2014		Elora 2015		Belwood 2015	
Fixed effects†	F-test	P-value	F-test	P-value	F-test	P-value
G	5.86	<.0001	7.93	<.0001	5.06	<.0001
SPADT	3.47	0.0637	4.35	0.0378	43.64	<.0001
G*SPADT	2.5	<.0001	1.65	0.0109	2.52	<.0001
Random effects‡	S^2	Se	S^2	Se	S^2	Se
BLOC	0.03	0.346^{ns}	0.00	.	0.00	.
IBLK(BLOC)	0.62	0.718	0.30	0.471^{ns}	0.73	0.9199^{ns}
observation(BLOC*G*SPADT)	13.73	1.2784***	14.00	1.1871***	22.38	1.8618***
Residual	0.94	0.000	0.94	0	0.96	0

† Genotype (G) main effect, SPAD timing (SPADT), and the interaction of G*SPADT

‡ Block (BLOC), and incomplete block (IBLK),

^{ns} Not significant; *, **, and *** are significant at 0.05, 0.01, and 0.001, respectively

Supplementary Table 7. Phenotypic (r_p) correlations among %Ndfa and other traits estimated in panel of 35 Heirloom or Standard-bred genotypes grown in Elora, Ontario, 2014.

	Days to Maturity (GDD)	$\delta^{13}C$ (‰)	Yield (kg ha^{-1})	Seed N content (mg)	Ndfa (%)
Days to flowering	0.59**	-0.04	-0.28	-0.13	0.24
Days to maturity		0.12	-0.02	0.08	0.10
$\delta^{13}C$			-0.21	0.15	-0.21
Yield				-0.21	-0.03
Seed N content					-0.04

* and ** are significant at 0.05 and 0.01, respectively. Significant r_p tested by the Student's t test.

Supplementary Table 8. Phenotypic (r_p) correlations among %Ndfa and other traits estimated in panel of Heirloom or Standard-bred genotypes grown in Elora, Ontario, 2015. The number of genotypes in each correlation is presented in the table.

	Days to Maturity (GDD)	$\delta^{13}C$ (‰)	Yield (kg ha-1)	Seed N content (mg)	Ndfa (%)
Days to flowering	0.76**	0.53**	-0.09	0.05	0.51**
number of genotypes	35	41	40	41	41
Days to maturity		0.37*	-0.13	-0.09	0.45**
number of genotypes		35	34	35	35
$\delta^{13}C$			0.11	0.07	0.45**
number of genotypes			40	41	41
Yield				-0.43763**	0.21
number of genotypes				40	40
Seed N content					-0.07
number of genotypes					41

* and ** are significant at 0.05 and 0.01, respectively. Significant r_p tested by the Student's t test.

Supplementary Table 9. Phenotypic (r_p) correlations among %Ndfa and other traits estimated in panel of Heirloom or Standard-bred genotypes grown in Belwood, Ontario, 2015. The number of genotypes in each correlation is presented in the table.

	Days to Maturity (GDD)	$\delta^{13}C$ (‰)	Yield (kg ha-1)	Seed N content (mg)	Ndfa (%)
Days to flowering	0.59**	0.11	-0.06	0.38	0.55**
	40	41	40	41	41
Days to maturity		0.48**	0.06	0.56**	0.37
		40	40	40	40
$\delta^{13}C$			0.17	0.56**	0.26
			40	41	41
Yield				-0.05	0.04
				40	40
Seed N content					0.30
					41

* and ** are significant at 0.05 and 0.01, respectively. Significant r_p tested by the Student's t test.
